# Staphylococcal Biofilm on the Surface of Catheters: Electron Microscopy Evaluation of the Inhibition of Biofilm Growth by RNAIII Inhibiting Peptide

**DOI:** 10.3390/antibiotics10070879

**Published:** 2021-07-20

**Authors:** Adilson de Oliveira, Luiza Pinheiro-Hubinger, Valéria Cataneli Pereira, Danilo Flávio Moraes Riboli, Katheryne Benini Martins, Letícia Calixto Romero, Maria de Lourdes Ribeiro de Souza da Cunha

**Affiliations:** 1 Department of Chemical and Biological Sciences, Biosciences Institute, UNESP—Universidade Estadual Paulista, Botucatu 18618-691, Brazil; adilson270193@gmail.com (A.d.O.); valeriacataneli@gmail.com (V.C.P.); danilo.riboli@ibb.unesp.br (D.F.M.R.); katheryne_bm@yahoo.com.br (K.B.M.); leticia.calixto@unesp.br (L.C.R.); mlrs.cunha@unesp.br (M.d.L.R.d.S.d.C.); 2 Department of Anatomic Pathology, Lauro de Souza Lima Institute, Bauru 17034-971, Brazil

**Keywords:** *Staphylococcus aureus*, coagulase-negative staphylococci, biofilm structure, quorum sensing, catheter, RIP, biofilm inhibition, scanning electron microscopy

## Abstract

*Staphylococcus aureus* and coagulase-negative staphylococci (CoNS) have become the main causative agents of medical device-related infections due to their biofilm-forming capability, which protects them from the host’s immune system and from the action of antimicrobials. This study evaluated the ability of RNA III inhibiting peptide (RIP) to inhibit biofilm formation in 10 strains isolated from clinical materials, including one *S. aureus* strain, two *S. epidermidis*, two *S. haemolyticus*, two *S. lugdunensis*, and one isolate each of the following species: *S. warneri*, *S. hominis*, and *S. saprophyticus*. The isolates were selected from a total of 200 strains evaluated regarding phenotypic biofilm production and the presence and expression of the *ica* operon. The isolates were cultured in trypticase soy broth with 2% glucose in 96-well polystyrene plates containing catheter segments in the presence and absence of RIP. The catheter segments were observed by scanning electron microscopy. The results showed inhibition of biofilm formation in the presence of RIP in all CoNS isolates; however, RIP did not interfere with biofilm formation by *S. aureus*. RIP is a promising tool that might be used in the future for the prevention of biofilm-related infections caused by CoNS.

## 1. Introduction

Medical device-related infections are associated with the capability of bacteria to adhere and attach to surfaces and to subsequently form a biofilm [1,2]. Biofilms are composed of communities of microorganisms enveloped by an extracellular matrix consisting of polysaccharides or proteins produced by the bacteria themselves, which remain adhered to abiotic or biotic surfaces [3,4]. The bacteria present inside the biofilm may have a different virulence and resistance phenotype in terms of gene transcription and growth rate [5]. Biofilms are considered one of the most important virulence factors in *S. aureus* and are the most important virulence factor in the majority of coagulase-negative staphylococci (CoNS), especially *S. epidermidis*, protecting them from the host’s immune system and from the action of antibiotics [5,6]. The composition of the biofilm matrix varies among staphylococcal strains; however, the main class of exopolysaccharides in staphylococcal biofilms is polysaccharide intercellular adhesin (PIA), which is synthesized by four proteins, IcaA, IcaD, IcaB, and IcaC, encoded by the *icaADBC* operon [7,8,9].

Biofilm formation depends on the combination and expression of a variety of genes, whose expression is influenced by environmental factors such as growth conditions, carbohydrate supplementation, sub-inhibitory concentrations of antimicrobials, and high osmolarity. The bacteria use a cellular communication system called quorum sensing (QS) for this regulation, which works as a population density sensor [10,11]. This communication is mediated by signaling molecules called autoinducers that regulate the behavior of bacteria in response to population density. When a small number of bacteria releases autoinducers into the environment, their concentration is too low to be detected. However, when the population density is high, the concentration of autoinducers reaches a threshold that induces cells to respond to stimuli, activating or repressing target genes. This system thus allows the bacteria to coordinate their behavior according to the environmental conditions, as well as the expression of virulence factors, biofilm formation, and others [12].

**Figure 1:**
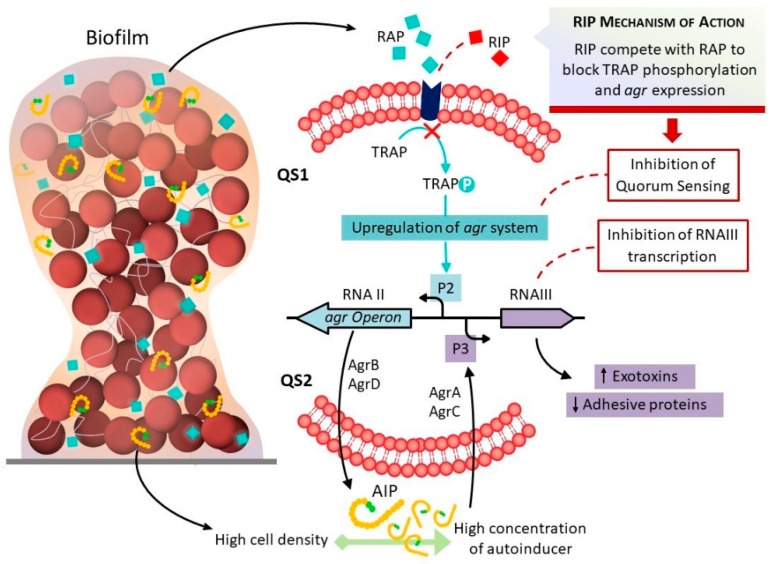
Schematic representation of *agr* control in staphylococci. As the bacteria multiply, RAP reaches a concentration threshold, inducing the phosphorylation of TRAP that triggers a poorly known mechanism for the synthesis of QSII, which consists of the products of the *agr* system. The *agr* system is composed of two divergent transcripts, RNAII and RNAIII. RNAII encodes AgrA, AgrC, AgrD, and AgrB. AgrD produces autoinducing peptide (AIP) that is processed by AgrB maturation and export. At a certain threshold concentration, AIP induces the phosphorylation of its receptor AgrC, and phosphorylated AgrA activates transcription from the P3 promoter, leading to the production of RNAIII. In addition to the presence of the RAP-TRAP system, there is also an antagonist, RIP, that can inhibit RNAIII activity. RIP competes with RAP to block TRAP phosphorylation, shutting down QS1 and *agr* expression.

Balaban et al. [13], studying a peptide (YSPWTNF-NH2) known as RNAIII inhibiting peptide (RIP), found that it can inhibit RNAIII activity by hindering the phosphorylation of TRAP, interrupting the expression of virulence factors, including biofilm formation (Figure 1). Studies conducted on catheter implanted animals in order to observe biofilm formation have shown that the injection of RIP resulted in low biofilm production and a low frequency of infections [14]. Other studies demonstrated that, in addition to being very effective in preventing device-related *Staphylococcus* infections, RIP also reduces bacterial load and can be useful for the treatment of infected wounds [15]. Thus, RIP might be in the future an interesting alternative to conventional antibiotics. However, according to some authors, the TRAP system does not participate in the activation of the *agr* system and hence plays no role in the pathogenesis of diseases caused by *Staphylococcus* [16,17].

Based on the above considerations, this study aimed to evaluate the effect of RIP on biofilm formation on catheters contaminated with *S. aureus* and CoNS by scanning electron microscopy (SEM) and the correlation with the expression of *ica* genes.

## 2. Results

The study comprised 200 *Staphylococcus* spp. strains, including 50 *Staphylococcus aureus* isolates and 150 CoNS isolates that belonged to six species. The 150 CoNS isolates were identified as follows: 50 *Staphylococcus epidermidis*, 7 *Staphylococcus lugdunensis*, 20 each of *Staphylococcus haemolyticus*, *Staphylococcus warneri*, and *Staphylococcus hominis* blood culture isolates, and 20 *Staphylococcus saprophyticus* isolated from the urine of patients with urinary tract infection.

### 2.1. Analysis of Biofilm Formation Using the Polystyrene Plate Adherence Method

Analysis of the 540 nm filter plates classified 41 (20.5%) of the 200 isolates as weakly adherent and 8 (4%) as strongly adherent, with a total of 49 (24.5%) positive isolates and 151 (75.5%) negative isolates (Table 1). Based on these results, we selected 10 biofilm-producing strains for the detection of the *ica* operon by the polymerase chain reaction (PCR). We verified gene expression by the reverse transcription-polymerase chain reaction (RT-PCR) technique and tested RIP to verify its effect on biofilm formation on catheter tips. The results were visualized using SEM and on polystyrene plates.

### 2.2. Detection of the Presence and Expression of icaADBC Genes by RT-PCR

The results of the detection and expression of the *ica* operon are shown in Table 2.

### 2.3. Analysis of the Inhibition of Biofilm Formation on Catheter Tips by RIP Using Scanning Electron Microscopy

For this test, we selected the following 10 strains that produced a biofilm in the polystyrene plate adherence test: two *S. epidermidis* (*S. epidermidis ADBC* and *S. epidermidis* *DBC*), one *S. saprophyticus*, two *S. haemolyticus* (*S. haemolyticus A* and *S. haemolyticus C*), one *S. hominis*, one *S. warneri*, two *S. lugdunensis* (*S. lugdunensis AD* and *S. lugdunensis_*), and one *S. aureus*. Two *S. epidermidis* strains were included as control: biofilm producer ATCC 35983 (weakly adherent) and non-biofilm producer ATCC 12228. Catheter tips in sterile trypticase soy broth (TSB) and RIP were used as negative controls.

**Figure 2:**
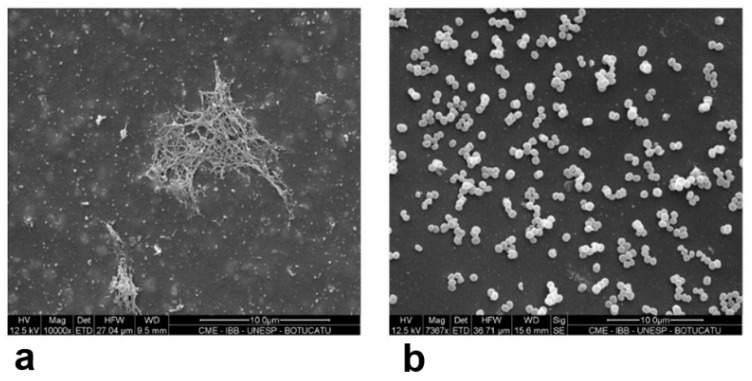
(**a**,**b**): SEM results for the positive control *S. epidermidis* ATCC 35983 (weakly adherent) (**a**) pure culture; (**b**) *S. epidermidis* ATCC 35983 isolate incubated with 200 µg/mL RIP. Scale bar (**a**,**b**): 10 µm.

The SEM results showed that adding 100 µg/mL of RIP was not sufficient to inhibit biofilm formation in *S. epidermidis* ATCC 35983 (positive control) and in the strongly adherent biofilm producer *S. saprophyticus* that expressed the *icaCDB* genes in the RT-PCR assay. In the catheter segment incubated with TSB alone, SEM analysis of *S. epidermidis* ATCC 35983 revealed a biofilm with a branched appearance, covering and connecting the cells (Figure 2). In the presence of 100 µg/mL RIP, we observed a very firm biofilm that occupied almost the entire catheter segment, with a large number of clustered cells attached to each other and few single cells.

**Figure 3:**
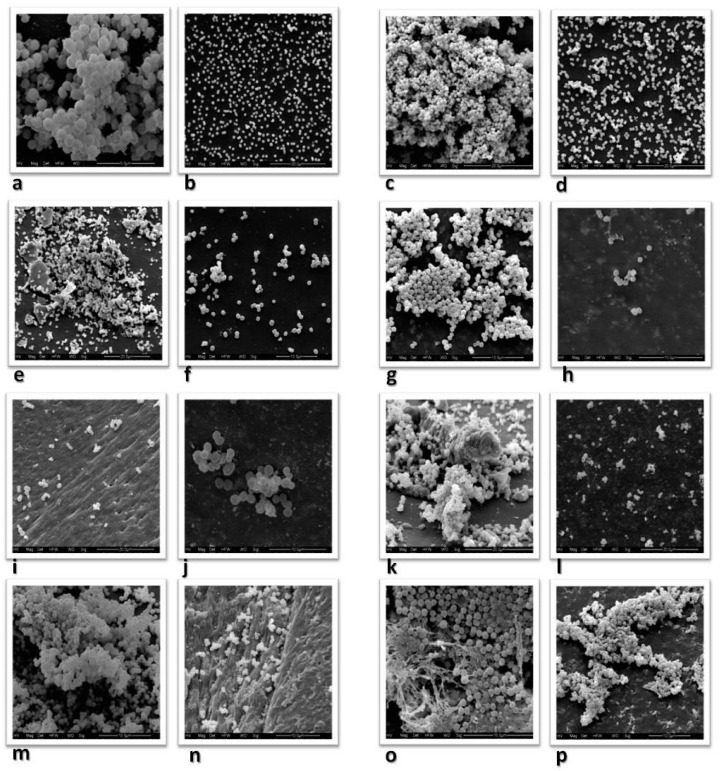
(**a**,**b**): SEM results for the same *S. saprophyticus* isolate: (**a**): pure culture; (**b**): *S. saprophyticus* isolate incubated with 200 µg/mL RIP. (**c**,**d**): *S. epidermidis ADBC* carrying the complete operon; (**c**): pure culture; (**d**): *S. epidermidis* incubated with 200 µg/mL RIP. (**e**,**f**): *S. epidermidis DBC* carrying the *icaDBC* genes; (**e**): pure culture; (**f**): *S. epidermidis* incubated with 200 µg/mL RIP. (**g**,**h**): *S. haemolyticus A* expressing only the *icaA* gene; (**g**): pure culture; (**h**): *S. haemolyticus* incubated with 200 µg/mL RIP. (**i**,**j**): SEM results for the same *S. hominis* isolate; (**i**): pure culture; (**j**): *S. hominis* incubated with 200 µg/mL RIP. (**k**,**l**): SEM results for the same *S. warneri* isolate; (**k**): pure culture; (**l**): *S. warneri* incubated with 200 µg/mL RIP. (**m**,**n**): SEM results for *S. lugdunensis*: (**m**): pure culture; (**n**): *S. lugdunensis* incubated with 200 µg/mL RIP. (**o**,**p**): SEM results for the same *S. aureus* isolate: (**o**): pure culture; (**p**): *S. aureus* incubated with 200 µg/mL RIP. Scale bars: (**a**,**b**): 10 µm; (**c**,**d**,**k**–**p**): 20 µm; (**e**–**h**): 10 µm; (**i**,**j**): 5 µm.

In view of the above results, the concentration of RIP was increased to 200 µg/mL in TSB+2% glucose. Figure 3a,b shows the SEM results for the same previously tested *S. saprophyticus* strain. Strongly adherent *S. saprophyticus* expressing the *icaDBC* genes produced a biofilm with a matrix that differed from the one observed in *S. epidermidis*. In this strain, the biofilm appeared as flocculent material enclosing and connecting the cells (Figure 3a). No biofilm formation was observed in the isolate incubated with RIP. The cells appeared dispersed and did not cover the entire surface of the catheter, a finding indicating a reduction in the number of bacteria (Figure 3b). SEM showed no alterations for the negative control, the catheter segment in TSB alone without inoculation or TSB alone with 200 µg/mL RIP.

Among the clinical *S. epidermidis* isolates, *S. epidermidis ADBC* expressed the full operon (*icaADBC*) in the RT-PCR assay, and SEM showed a very complex biofilm coating a large portion of the catheter segment, with the observation of a large number of bacteria (Figure 3c). In the isolate incubated with 200 µg/mL of RIP, the cells were dispersed, and a smaller number coated the catheter segment, considerably reducing biofilm formation (Figure 3d). In the case of the second strongly adherent *S. epidermidis CDB* isolate in the polystyrene plate test, RT-PCR revealed no expression of the *icaA* gene, but the expression of *icaCDB* genes was detected. Although present on most parts of the catheter, higher biofilm concentrations were observed at its ends during the study, with cells adhered to each other forming large and small clusters (Figure 3e). There was a decrease in biofilm formation in the isolate incubated with RIP, with the observation of dispersed cells and smaller quantities (Figure 3f).

Among the *S. haemolyticus* isolates, *S. haemolyticus A* was strongly adherent in the phenotypic test, but RT-PCR only revealed the expression of the *icaA* gene. The SEM analysis showed a firm biofilm with large amounts of polysaccharides connecting and enveloping the cells (Figure 3g). No biofilm formation was observed in the isolate incubated with RIP. In fact, there was a considerable decrease in the number of bacteria coating the catheter (Figure 3h). The other *S. haemolyticus C* isolate was weakly adherent in the phenotypic test, tested positive by PCR only for the *icaCD* genes, and expressed only the *icaC* gene in the RT-PCR assay. In SEM analysis, the biofilm was mainly present at the ends and did not cover the entire surface of the catheter. Incubation of this isolate with RIP inhibited biofilm formation and considerably decreased the number of bacteria coating the catheter.

Only one *S. hominis* isolate was positive/weakly adherent in the phenotypic test, which did not express any of the *ica* operon genes. The SEM analysis showed small cell clusters, mainly at the ends of the catheter (Figure 3i). No biofilm formation was observed on the catheter incubated with RIP, and there was an even smaller number of bacteria adhering at single points of the catheter (Figure 3j).

The *S. warneri* isolate was weakly adherent in the plate test and did not express any of the *ica* operon genes. The SEM analysis showed a large number of bacteria almost covering the entire surface of the catheter. The biofilm was well-structured and firm, with cells attached to each other by a “gelatinous” substance, possibly proteins. However, this finding was mainly noted at the ends of the catheter (Figure 3k). In the isolate incubated with RIP, SEM showed dispersed cells, with no formation of a biofilm and a visible decrease in the number of bacteria on the surface of the catheter (Figure 3l).

Two *S. lugdunensis* isolates were studied. *S. lugdunensis AD* was strongly adherent in the plate test and expressed the *icaAD* genes in the RT-PCR assay. The SEM analysis showed a well-structured biofilm similar to that produced by the *ica*-positive *S. epidermidis* isolate. However, the biofilm was more frequently located at the ends of the catheter. In the sample incubated with RIP, the cells appeared isolated, with no biofilm formation in any portion of the catheter. The other *S. lugdunensis* isolate was weakly adherent in the phenotypic test and did not express any of the *ica* operon genes in the RT-PCR assay, although the *icaA* gene was detected by PCR. The SEM analysis showed a large number of bacteria covering almost the entire surface of the catheter, exhibiting a biofilm with a large cluster of cells attached by a “gelatinous” substance, similar to what was previously described in the absence of *ica* expression (Figure 3m). When incubated with RIP, SEM showed no biofilm formation (Figure 3n). There was a considerable decrease in the number of bacteria on the surface of the catheter, with cells only being found at its ends. In *S. epidermidis* ATCC 12228 incubated with TSB alone (negative control), SEM showed a small number of single bacteria covering the surface of the catheter and no biofilm formation. A reduction in the number of bacteria was also observed when the isolate was incubated with RIP.

The *S. aureus* isolate studied was strongly adherent in the polystyrene plate test, and PCR detected the complete *icaADBC* operon. RT-PCR revealed the expression of all four genes. The SEM analysis showed a biofilm with large cell clusters and an “elastic” structure, probably composed of polysaccharides that visibly covered the cells (Figure 3o). Incubation with RIP did not result in complete biofilm elimination in this species. We found smaller cell clusters in biofilm in different parts of the surface of the catheter (Figure 3p).

### 2.4. Analysis of the Inhibition of Biofilm Formation by RIP Using Polystyrene Plates 

In this assay, the isolates of the different CoNS species tested did not form a biofilm in the presence of 200 µg/mL RIP. Only the *S. aureus* isolate tested positive (strongly adherent), showing excellent agreement with the SEM results. Table 3 shows the percentage of inhibition of biofilm formation in the presence of RIP.

## 3. Discussion

The QS systems in *Staphylococcus* have a major effect on how successful the pathogen is during infection by using physiological and virulence control mechanisms such as biofilm production. We evaluated biofilm formation using SEM in isolates of *S. epidermidis*, *S. saprophyticus*, *S. haemolyticus*, *S. hominis*, *S. warneri*, *S. lugdunensis*, and *S. aureus* cultured with TSB alone + 2% glucose and TSB + 2% glucose with RIP. According to Balaban et al. [13,18], this peptide (YSPWTNF-NH2) can inhibit RNAII activity, inhibiting the phosphorylation of TRAP and interfering with the QS system. Korem et al. [19] and Balaban et al. [20] reported that the mechanism of action of RIP differs from that of the common antibiotics, inhibiting bacterial cell-cell communication and preventing adhesion and the expression of virulence factors. Genomic studies have shown that in the absence of TRAP phosphorylation, the bacteria lose their ability to produce toxins, adhere to surfaces, and form biofilms. In view of these results, the concentration of RIP was increased to 200 µg/mL in TSB + 2% glucose. The SEM results showed that this concentration prevented biofilm formation in all CoNS isolates studied. In the presence of RIP, single cells were observed in both strongly adherent and weakly adherent strains that expressed the *icaA* gene and other *ica* operon genes, or that did not express any of these genes in the RT-PCR assay. Similar results have been reported by other authors [14] who demonstrated the efficiency of RIP in inhibiting staphylococcal biofilm formation. Another interesting fact we observed in the catheter segments with RIP is that, in addition to preventing biofilm formation, the peptide considerably decreased the number of bacteria, including the non-biofilm producer ATCC 12228. Simonetti et al. [15] reported that RIP could be very useful in treating infected wounds as it reduces bacterial load and may therefore be a future alternative to conventional antibiotics.

For the study and comparison of biofilm structure in *S. epidermidis* we analyzed two isolates by SEM. One isolate was strongly adherent in the phenotypic test and expressed the complete *icaADBC* operon in the RT-PCR assay. The other isolate was strongly adherent and expressed the *icaDBC* genes. The isolate carrying the full operon, *S. epidermidis ADBC*, exhibited a well-structured biofilm consisting of a large cluster of cells attached to each other and surrounded by polysaccharides, which covered almost the entire catheter with the slime covering and connecting the cells. On the other hand, the isolate that expressed the *icaDBC* genes but not *icaA*, *S. epidermidis DBC*, had a less structured biofilm whose characteristics differed from those described for the other isolate. The cocci appeared to be attached to each other but in small clusters and over an amorphous surface. This biofilm configuration might be explained by the absence of the *icaA* gene that reduced the amount of polysaccharide, or may have occurred by other mechanisms.

The matrix of the biofilm formed by the strongly adherent *S. saprophyticus* isolate expressing the *icaDBC* genes differed from that seen in the *ica*-positive *S. epidermidis* isolate. We observed a large number of cells attached to each other through substances produced by the bacteria. Instead of the matrix covering the cells, we found a flocculent material that represents dehydrated biofilm materials, according to Marrie and Costerston [21]. These results suggest the presence of genes encoding other substances involved in the formation of the biofilm matrix in *S. saprophyticus*.

Regarding the strongly adherent *S. haemolyticus A* isolate that expressed only the *icaA* gene in RT-PCR analysis, the bacteria were present in small clusters connected by polysaccharide-like strands, as also observed in the *ica*-positive *S. epidermidis* isolates; however, the matrix did not contain large amounts of polysaccharides. Fredheim et al. [22], studying the formation and structure of *S. haemolyticus* biofilms, reported that only two of 72 isolates carried the *ica* operon genes and were positive for *icaD*. However, most of the isolates tested in the phenotypic polystyrene plate adherence test produced a biofilm in the absence of the *ica* operon. With respect to the components of the biofilm matrix in *S. haemolyticus*, the authors reported that proteins and extracellular DNA are of functional importance for the steps of biofilm accumulation and formation, while PIA (Polysaccharide Intercellular Adhesin) plays only a small role, a fact that may explain the results found in our study of this species.

In *S. hominis* and *S. warneri*, SEM showed a biofilm whose structural characteristics were similar in the two species. *Staphylococcus hominis* formed small structures found at the ends of the catheter, while *S. warneri* produced very firm structures containing a larger number of adhering bacteria, also located at the ends of the catheter. The similar results obtained for the two species suggest that biofilm formation does not depend on the expression of the *icaA* gene in these species. According to Kini et al. [23], *S. warneri* is associated with many endocarditis cases. This fact might be explained by the components of the biofilm matrix in these species that have an affinity for biotic surfaces.

*Staphylococcus lugdunensis AD* was strongly adherent in the plate test and expressed the *icaAD* genes in the RT-PCR assay. The SEM analysis showed a highly consistent biofilm with structural characteristics similar to those observed in *S. epidermidis*; however, the biofilm was more frequently found at the ends of the catheter. For the isolate that was weakly adherent in the plate test and expressed none of the *ica* operon genes in the RT-PCR assay, SEM showed a large number of bacteria covering almost the entire surface of the catheter and exhibiting a biofilm consisting of a large cell cluster. According to Frank and Patel [24], many types of infection caused by *S. lugdunensis*, including native valve endocarditis, prosthetic joint infection, and intravascular catheter-related infection, are associated with biofilm formation. However, studies performed by the authors showed that, in most biofilms of this species, the polysaccharide synthesized by the *icaADBC* operon is not the main matrix component, suggesting that the majority of biofilms consist of proteins. Experiments using different chemical substances in the growth medium for biofilm formation suggested that the conditions of the medium can influence biofilm components and structure. Although *S. lugdunensis* has emerged as an important pathogen implicated in infections, data on the biofilm-forming potential of this species are scarce. The results found in this study are of great importance for the characterization of the pathogenic potential of this species.

The strongly adherent *S. aureus* that carried the *icaADBC* genes and expressed the four genes in the RT-PCR assay exhibited a very firm cell cluster covered by a visible “elastic” structure, which was probably composed of polysaccharides. Sun et al. [25] investigated the properties and architecture of *S. aureus* biofilms on polystyrene plates using SEM. The results showed that the biofilm consisted of multilayered cell clusters with a three-dimensional architecture, similar to our findings. However, incubation of the *S. aureus* isolates with RIP resulted in a very discrete reduction. We found cell clusters in the biofilm structure and on several parts of the catheter surface. An increase in the concentration of the peptide is probably necessary to completely inhibit biofilm formation in this species.

To confirm the effect of RIP for preventing biofilm formation, we performed the same comparison of the isolates using polystyrene plates, and the results were similar to the microscopy findings. RIP inhibited biofilm formation in all CoNS isolates but not in *S. aureus*. *S. aureus* isolates were strongly adherent in the plate test, even in the presence of the peptide.

The SEM analysis revealed a structure with polysaccharide characteristics in the biofilm-producing isolates that expressed the full operon or the *icaA* gene. This analysis, along with the polystyrene plate adherence test, confirmed the inhibition of biofilm formation by RIP in CoNS species. Our study demonstrated that, in addition to being effective in inhibiting biofilm formation by CoNS species, RIP also reduced the growth of these microorganisms on catheter surfaces. Taken together, these findings suggest the possibility of using this substance in the future as an alternative to conventional antibiotics in the control of catheter-related infections. More studies are necessary to confirm the antimicrobial activity of RIP in *Staphylococcus* spp.

As a limitation of this study, although SEM images are useful to illustrate particular aspects of the biofilm morphology, they do not provide information on the chemical composition of the extracellular matrix, which should be explored using other approaches. Further studies using confocal laser microscopy are necessary to obtain more defined images. In addition, to confirm the data on bacterial growth reduction, it is important to quantify colony-forming units in the presence of RIP and to determine the minimum inhibitory concentration of this peptide for *Staphylococcus* spp.

## 4. Materials and Methods

### 4.1. Strains

For the selection of biofilm-producing strains, we studied 200 *Staphylococcus* spp. isolates, including 50 *S. aureus* isolates and 150 CoNS isolates. The CoNS strains included 50 *S. epidermidis* isolates; 20 isolates each of *S. haemolyticus*, *S. warneri*, and *S. hominis*; 7 *S. lugdunensis* blood culture isolates, and 20 *S. saprophyticus* isolated from the urine of patients with urinary tract infection treated at the University Hospital of the Botucatu Medical School (FMB). Due to the difficulty of isolating *S. lugdunensis* from human clinical samples, we used 13 *S. lugdunensis* isolates from goat milk. The following international reference strains were included as controls: *S. epidermidis* ATCC 12228 and *S. aureus* ATCC 33591 (negative control), and *S. epidermidis* ATCC 35983 and *S. aureus* ATCC 29213 (positive control).

The present study was approved by the Research Ethics Committee of the Botucatu Medical School, São Paulo State, Brazil (Approval number 3783–2011).

### 4.2. Identification of Microorganisms

After their growth on blood agar plates, the isolates were submitted to Gram staining for the analysis of purity, morphology, and specific staining. The microorganisms were identified as suggested by Koneman et al. [26].

### 4.3. Identification of Staphylococcus

For the identification of *Staphylococcus* strains, initial screening was performed by the catalase test, followed by the tube coagulase test to differentiate *S. aureus* from CoNS strains using the criteria proposed by Koneman et al. [26]. The CoNS species were confirmed by genotyping using conserved primer sequences adjacent to the 16S and 23S genes, following the internal transcribed spacer (ITS)-PCR technique described by Couto et al. [27]. DNA was extracted from the isolates identified as CoNS with the Illustra Kit (GE Healthcare, Salt Lake, UT, United States) according to the manufacturer’s instructions. CoNS were genotyped using the following primers that target conserved sequences adjacent to the 16S and 23S genes: G1 “GAAGTCGTAACAAGG” 16S and L1 “CAAGGCATCCA CCGT” 23S. The efficiency of the amplification was monitored by electrophoresis on 3% MetaPhor agarose prepared in 1× TBE buffer and stained with SYBR Safe. The following international reference strains were used as controls: *S. epidermidis* (ATCC 12228), *S. epidermidis* (ATCC 35983), *S. haemolyticus* (ATCC 29970), *S. hominis* (ATCC 27844), *S. hominis* subsp. novobiosepticus (ATCC 700237), *S. lugdunensis* (ATCC 700328), *S. saprophyticus* (ATCC 15305), and *S. warneri* (ATCC 10209).

### 4.4. Analysis of Biofilm Formation by the Polystyrene Plate Adherence Method

The method for detecting biofilm production on culture plates described by Christensen et al. [28] was used, with modifications proposed by Oliveira and Cunha [29]. This method is based on the determination of spectrophotometric optical density (OD) of the adherent material produced by the bacteria. International reference strains used as positive (*S. epidermidis* ATCC 35983) and negative controls (*S. epidermidis* ATCC12228) and sterile TSB were included in all tests. OD readings were obtained in an ELISA reader (Multiskan EX, Labsystems, Midland, Canada)) using a 540 nm filter. The isolates were classified as negative when the cut-off value corresponded to the non-adherent value (≤0.111) and as positive when the cut-off value corresponded to the weakly adherent (>0.111 or ≤0.222) or strongly adherent (>0.222) value. These cut-offs values were established by Oliveira and Cunha [29].

### 4.5. Nucleic Acid Extraction and Amplification (PCR)

The parameters and primers described by Arciola et al. [30,31] and Rohde et al. [32] (Table 4) were used for nucleic acid extraction and PCR. International reference strains were included in all reactions: *S. epidermidis* ATCC 35983 and *S. aureus* ATCC 29213 (biofilm producer) as positive control and *S. epidermidis* ATCC 12228 and *S. aureus* ATCC 33591 (non-biofilm producer) as a negative control.

### 4.6. Visualization of the Amplified Products

The efficiency of the amplifications was monitored by electrophoresis of the reaction on 2% agarose gel in 0.5× Tris-borate-EDTA (TBE) buffer stained with SYBR Safe (Invitrogen, Carlsbad, CA, United States). The size of the amplified products was compared to a standard 100 bp ladder, and the gels were photographed under UV transillumination.

### 4.7. RNA Extraction and Gene Expression Analysis of the Ica Operon by RT-PCR

Total RNA was extracted from *Staphylococcus* spp. strains cultured on blood agar, individually inoculated in brain-heart infusion (BHI) broth, and incubated for 24 h at 37 °C. The Illustra RNAspin Mini RNA kit was used for RNA extraction following the manufacturer’s instructions. For this, 200 μL of the *Staphylococcus* culture was transferred to a sterile 1.5 mL Eppendorf tube. After centrifugation at 10,000× *g* for 1 min, the supernatant was removed, 100 μL TE buffer containing 2 mg/mL of lysozyme was added, and the mixture was incubated for 10 min at 37 °C. For cell lysis, 350 μL of RA1 buffer and 3.5 μL of β-mercaptoethanol were added. The solution was then applied to RNAspin Mini Filter units, and the filters were centrifuged at 11,000× *g* for 1 min. The filters were discarded after centrifugation. Next, 350 μL of 70% ethanol was added to the filtered solution for adjustment of the ligation conditions. The filtered solution was then transferred to an RNAspin Mini Column and centrifuged at 8000× *g* for 30 s. For ligation to the membrane, 350 μL of membrane desalting buffer was added, and the solution was centrifuged at 11,000× *g* for 1 min. The samples were washed in two steps. For the first cell wash, 600 μL of RA3 buffer was added to the spin column, and the column was centrifuged at 11,000× *g* for 1 min. For the second cell wash, 250 μL of RA3 buffer was added to the spin column, and the column was centrifuged at 11,000× *g* for 2 min. The column was then transferred to a new 1.5 mL Eppendorf tube for RNA elution. For this purpose, 45 μL of RNase-free water and 5 µL of RNA guard were added, and the tube was centrifuged at 11,000× *g* for 1 min. For the total elimination of any DNA residues, the samples were treated with DNAse (2 μL of buffer solution and 2 μL of DNAse) for 1 h at 37 °C. The DNAse enzyme was blocked by the addition of 2 μL of DNAse Stop Solution and incubation for 10 min at 65 °C. The RNA was immediately stored at −80 °C.

### 4.8. cDNA Synthesis 

Two mixtures were prepared (Mix 1 and Mix 2). Mix 1 contained 14 μL of RNA (already aliquoted and treated with DNase), 1 μL of Random Primer, 1 μL of dNTP, and 4 μL of nuclease-free water (extraction kit). Mix 2 contained 4 μL of 5× First-Strand Buffer, 1 mL of DTT (0.1 M), and 1 μL of SuperScript III (200 U/μL). Mix 1 was incubated in a thermal cycler for 5 min at 65 °C, removed from the thermal cycler, and immediately transferred to a gel for approximately 5 min. Mix 2 (volume of 6 μL) was then added, and the sample was again placed in the thermal cycler, continuing the program. The cDNA was then frozen at −80 °C.

### 4.9. RT-PCR

The RT-PCR program of the thermal cycler was used, which consisted of cycles at 65 °C for 5 min, 25 °C for 5 min, 50 °C for 60 min, 70 °C for 15 min, and finishing at 20 °C. After PCR of the internal control (16S RNA-encoding gene, 16S_F_ CCT ATA AGA CTG GGA TAA CTT CGG G and *16S_R_* CTT TGA GTT TCA ACC TTG CGG TCG) for control of the extraction procedure, RT-PCR, and efficiency of the DNAse enzyme, we performed PCR to verify the expression of the *ica* operon genes using the primers described in Table 4. The amplified products were visualized by electrophoresis as described in item 4.6.

### 4.10. Analysis of the Inhibition of Biofilm Formation on Catheter Tips by RIP Using Scanning Electron Microscopy

The effect of RIP on biofilm formation was evaluated in 10 isolates selected based on the results of the detection of the *ica* operon. Gene expression was analyzed by the RT-PCR technique and biofilm formation by the polystyrene plate adherence test using segments of sterilized catheters. The biofilm-producing strains were first isolated in BHI broth, and 10^8^ CFU were transferred to conical tubes (Falcon, Corning) containing 2 mL of TSB growth medium with 2% glucose. Some of the tubes contained pure culture, and others also contained 200 µg/mL RIP and a 0.5 cm segment of a Vygon umbilical catheter (Product code: 1270.04; 0.8 mm × 1.5 mm in diameter). These tubes were incubated under constant stirring at 100 rpm/37 °C for 48 h to allow bacterial growth and biofilm formation. After this period, the catheter segments were removed and immersed in a 2.5% glutaraldehyde solution; fixed in an increasing alcohol series (15%, 30%, 50%, 70%, 90%, and 100%) for 15 min each; dried in a vacuum centrifuge for 5 min; sputtered with gold and visualized under a scanning electron microscope to confirm biofilm formation in the presence and absence of RIP. *S. epidermidis* ATCC 12228 was used as negative control and *S. epidermidis* ATCC 35983 as a positive control. All tests and the SEM analysis were performed in duplicate.

### 4.11. Analysis of the Inhibition of Biofilm Formation by RIP Using Polystyrene Plates

For this analysis, we employed the method of biofilm formation on cell culture plates proposed by Christensen et al. [28] and modified by Oliveira and Cunha [29] using the 10 isolates selected for the previous test. The biofilm-producing strains were first isolated in BHI broth, and 10^8^ CFU were transferred to conical tubes (Falcon, Corning) containing 2 mL of TSB growth medium with 2% glucose. Some of the tubes contained pure culture, and others also contained 200 µg/mL RIP. The same procedures as used in the polystyrene plate adherence test were then followed. *S. epidermidis* ATCC 12228 was used as negative control and *S. epidermidis* ATCC 35983 as a positive control. All isolates were tested four times to ensure reproducibility of the test. The OD was determined with an ELISA reader (Multiskan EX, Labsystems, Midland, Canada) using 540 m filters.

## Figures and Tables

**Table 1:** Identification of biofilm-producing *Staphylococcus* spp. isolates using the polystyrene plate adherence method.

Species	Plate Adherence Test		
	SA	WA	NA
*S. aureus*, % (*n*)	12.5 (1)	31.7 (13)	23.8 (36)
*S. epidermidis*, % (*n*)	50.0 (4)	22.0 (9)	24.5 (37)
*S. saprophyticus*, % (*n*)	12.5 (1)	29.3 (12)	4.6 (7)
*S. haemolyticus*, % (*n*)	12.5 (1)	4.9 (2)	11.3 (17)
*S. hominis*, % (*n*)	-	2.4 (1)	12.6 (19)
*S. warneri*, % (*n*)	-	7.3 (3)	11.3 (17)
*S. lugdunensis*, % (*n*)	12.5 (1)	2.4 (1)	11.9 (18)
Total number of isolates, % (*n*)	100 (8)	100 (41)	100 (151)

WA: weakly adherent; SA: strongly adherent; NA: non-adherent.

**Table 2:** Detection of the *ica* genes (*ADBC* genes) gene expression analyzed using RT-PCR and biofilm formation detected by the polystyrene plate adherence method in the isolates selected for the study.

Species	PCR	RT-PCR	Plate Adherence Test
*S. aureus*	*ADBC*	*ADBC*	SA
*S. epidermidis*	*ADBC*	*ADBC*	SA
*S. epidermidis*	*ADBC*	*DBC*	SA
*S. saprophyticus*	*ADBC*	*DBC*	SA
*S. haemolyticus*	*ADBC*	*A*	SA
*S. haemolyticus*	*CD*	*C*	WA
*S. hominis*	*ADC*	-	WA
*S. warneri*	*DC*	-	WA
*S. lugdunensis*	*AD*	*AD*	SA
*S. lugdunensis*	*A*	-	WA

Plate adherence test result: WA: weakly adherent; SA: strongly adherent. Results of the detection and expression of the *ica ADBC* operon: *icaA* (*A*), *icaD* (*D*), *icaB* (*B*), and *icaC* (*C*).

**Table 3:** Quantification of biomass and percentage of inhibition of biofilm formation by RIP using polystyrene plates: TSB alone + 2% glucose and TSB + 2% glucose with 200 µg/mL RIP.

Strains	Plate Adherence Test	OD TSB Alone + 2% Glucose	OD TSB + 2% Glucose with 200 µg/mL RIP	Biofilm Inhibition (%)
*S. saprophyticus _ACDB_*	SA	0.315	0.109	65.4
*S. epidermidis _ACDB_*	SA	0.319	0.110	65.5
*S. epidermidis _CDB_*	SA	0.236	0.055	76.7
*S. haemolyticus _A_*	SA	0.261	0.074	71.6
*S. hominis*	WA	0.118	0.077	34.7
*S. warneri*	WA	0.176	0.049	72.1
*S. lugdunensis _AD_*	SA	0.287	0.042	85.4
*S. aureus _ACDB_*	SA	0.441	0.398	9.7
*S. epidermidis* _ATCC 35983_	WA	0.193	0.037	80.8
*S. epidermidis* _ATCC 12228_	NA	0.077	0.055	28.6

Results of the detection and expression of the *ica ADBC* operon: *icaA* (*A*), *icaD* (*D*), *icaB* (*B*), and *icaC* (*C*). *S. epidermidis* ATCC 35983: positive control; *S. epidermidis* ATCC 12228: negative control. OD: Optical density was determined with an ELISA reader (Multiskan EX, Labsystems) using 540 nm filters. Plate adherence test result: WA: weakly adherent; SA: strongly adherent; NA: non-adherent.

**Table 4:** Oligonucleotides used for the detection of the *icaA*, *icaB*, *icaC*, and *icaD* genes.

Primer	5′ to 3′ Nucleotide Sequence	Amplified Product (bp)	Reference
*icaA_F_*	TCT CTT GCA GGA GCA ATC AA	187	[30]
*icaA_R_*	TCA GGC ACT AAC ATC CAG CA		
*icaA_F_*	TGG CTG TAT TAA GCG AAG TC	669	[32]
*icaA_R_*	CCT CTG TCT GGG CTT GAC C		
*icaB_F_*	CTG ATC AAG AAT TTA AAT CAC AAA	302	[31]
*icaB_R_*	AAA GTC CCA TAA GCC TGT TT		
*icaC_F_*	TAA CTT TAG GCG CAT ATG TTT	400	[31]
*icaC_R_*	TTC CAG TTA GGC TGG TAT TG		
*icaD_F_*	ATG GTC AAG CCC AGA CAG AG	198	[30]
*icaD_R_*	CGT GTT TTC AAC ATT TAA TGC AA		

F: forward; R: reverse.

## Data Availability

The data presented in this study are contained within the article.
